# Global trends of acupuncture clinical research on analgesia from 2010 to 2023: a bibliometric and visualization analysis

**DOI:** 10.3389/fneur.2024.1368988

**Published:** 2024-04-11

**Authors:** Zhi-Qiang Li, Xue-Feng Wang, Cao Feng, Yu-Tong Fei, Jian-Ping Liu

**Affiliations:** ^1^Centre for Evidence-based Chinese Medicine, Beijing University of Chinese Medicine, Beijing, China; ^2^The National Research Center in Complementary and Alternative Medicine (NAFKAM), Department of Community Medicine, Faculty of Health Science, UiT the Arctic University of Norway, Tromsø, Norway

**Keywords:** acupuncture, analgesia, bibliometric analysis, hotspots, visualization analysis

## Abstract

**Objective:**

Acupuncture, acknowledged as a potent non-pharmacological therapy, is frequently employed to alleviate pain. Despite its widespread use, there has been a lack of overarching bibliometric analysis of clinical research on acupuncture analgesia. We aimed to summarize current patterns, hotspots, and development trends in this field through bibliometric analysis.

**Methods:**

This study evaluates academic publications retrieved from the Web of Science database (2010.01–2023.09) concerning acupuncture analgesia in clinical settings. All primary and secondary studies on humans were included. To track global developmental trends, we employed several software for analyzing annual publication volumes, countries/regions, institutions, authors, cited authors, journals, cited journals, references, and keywords and to draw collaborative networks and reference co-citation network maps.

**Results:**

The final search encompassed 7,190 relevant studies, including 1,263 randomized controlled trials (RCTs) and 1,293 systematic reviews and meta-analyses. The results indicated a gradual increase in the number of annual publications on acupuncture analgesia in clinical practice. Among countries and institutions, China (2,139) and Chengdu University of Traditional Chinese Medicine (258) ranked first. Liang FR (89 articles) was the most prolific author, while MacPherson H (604) was the most cited author. MEDICINE (455) was the most productive journal, and Pain (2,473/0.20) ranked first in both the frequency and centrality of cited journals. Notably, the most frequently cited reference was a systematic review of individual patient data on acupuncture carried out for chronic pain that was published by Vickers Andrew J in 2012 (156). Burst analysis identified frontier research areas for 2010–2020, encompassing network meta-analysis, case reports, dry needling, lumbar disc herniation, cancer, post-herpetic neuralgia, insomnia, and bibliometric analysis.

**Conclusion:**

This study outlines current trends and potential future research hotspots in clinical acupuncture analgesia over the past decade. Findings emphasize the necessity for enhanced international collaboration to improve research output and translation.

## Introduction

Pain is a common, intricate condition marked by the body's physiological and psychological responses to noxious stimuli, significantly impacting the patient's quality of life. Without appropriate treatment, persistent chronic pain may lead to complications such as hypochondria, depression, insomnia, and decreased appetite (1). The quest for effective pain treatments has been a subject of growing interest in healthcare practitioners. While pharmacotherapy, psychological approaches, and placebos are employed in clinical pain management, studies revealed limited efficacy and potential for substance abuse, including cocaine and opioids (2–4). Thus, a safe and efficacious alternative treatment is imperative.

Acupuncture, a potent non-pharmacological therapy in complementary and alternative medicine, is widely used for managing various diseases, particularly in clinical healthcare settings (5). Numerous studies have demonstrated the efficacy and safety of acupuncture in treating acute and chronic pain, such as shoulder pain (6), low back pain (7), and migraine (8), significantly enhancing the patient's quality of life. The mechanism of acupuncture includes various physiological pathways (4), including the release of endorphins and other neurotransmitters, which play a vital role in the analgesic process. This mechanism is akin to the body's natural pain-relieving process and is free of the side effects of many medications. With minimal side effects such as minor bleeding, bruising, dizziness, or fainting, notably less severe than those associated with non-steroidal anti-inflammatory drugs (NSAIDs) and opioids (e.g., gastric ulcers, constipation, respiratory depression, and addiction) (2–4). Furthermore, acupuncture stimulates the body's immune and circulatory systems, further enhancing its analgesic effects, which makes acupuncture a viable, low-risk option for pain management.

Acupuncture analgesia has been widely studied and applied in clinical practice. Consequently, understanding research trends and hotspots in this field is crucial for researchers. Bibliometrics, an interdisciplinary field using mathematical statistics for quantitative analysis of literature and knowledge dissemination, focuses on metrology characteristics to explore the dynamic characteristics of science and technology (9). Overcoming the subjective limitations of traditional reviews, bibliometrics facilitates the identification of crucial research directions, an understanding of developmental trends, and the recognition of hotspots in medical fields. More importantly, visualized maps provide valuable insights, aiding in the identification of established and emerging research areas for guiding clinical practice and decision-making (10). Since 2010, bibliometric studies on acupuncture analgesia have been emerging, covering various types of pain (6–8). However, these studies are limited to a single pain field, and there is no bibliometric analysis on acupuncture analgesia in clinical practice. Therefore, we aimed to use bibliometric analysis to encapsulate the progress and results of acupuncture analgesia in clinical research from 2010 to 2023, demonstrating the research trends and development trajectories and evaluating the analgesic effect of acupuncture, providing better guidance for research and clinical practice.

## Materials and methods

### Data sources and search strategies

All data were sourced from the Science Citation Index Expanded (SCI-E) within the Web of Science Core Collection (WoSCC) databases (11). Relevant publications were systematically identified through a comprehensive search and extraction process. To avoid omissions, we employed synonyms for “acupuncture,” “pain,” and “clinical” topics to collect data. The search time was from 1 January 2010 to 1 September 2023, resulting in the identification of 8,118 records. Subsequently, we refined the dataset to include only articles and reviews (*n* = 7,888), restricted the language to English only (*n* = 7,730), and excluded animal experiments involving rats, mice, and dogs (*n* = 523). Through manual scrutiny, we further eliminated redundant publications (*n* = 17). Finally, a total of 7,190 records were retained for analysis. For transparency, the specific retrieval strategy and flow chart are shown in Supplementary Table 1, Supplementary Figure 1.

### Data analysis

By using the mainstream bibliometric analysis tools, including CiteSpace (12, 13) (V6.2.R4, Drexel University, Philadelphia, PA, USA), VOSviewer (14–16) (Version1.6.19, Centre for Science and Technology Studies, Leiden University, Leiden, Netherlands), and R software (version 3.6.3), we conducted a bibliometric analysis (17). The integration of both CiteSpace and VOSviewer was essential for robust analysis, considering variations in algorithms and analysis thresholds (12–16).

VOSviewer is a powerful tool used for constructing and visualizing bibliometric networks based on countries/regions, institutions, journals, co-cited journals, and references of the publications. The visualized map contains three key elements, namely, size, distance, and colors. Nodes representing different entities, such as countries, institutions, and journals, vary in size based on the number of publications. In addition, the distance between nodes indicates their relatedness, with closer proximity signifying stronger links. Thicker links and shorter distances denote closer cooperation. Different colors signify distinct clusters.

CiteSpace presents the relationship between the literature on scientific knowledge maps that enable scholars to sort past research paths and depict prospects, allowing researchers to have a clearer view of the trends and directions in their research fields. The CiteSpace network map encompasses nodes, links, and colors. Node size corresponds to the number of publications with different elements, such as authors and cited authors. The larger the node, the greater the number of publications or citations in different subjects or domains. A purple circle on the outermost layer of a node denotes high centrality (node centrality > 0.1), highlighting articles with a significant impact and serving as key turning points in the field. Links between nodes represent their co-occurrence in the same publication, with thinner links indicating less frequent co-occurrence. In addition, the color of the links represents the year of first appearance, with warmer colors denoting more recent years. CiteSpace conducts analyses based on authors, co-cited authors, co-cited references, keyword citation bursts for forecasting the possible hotspots, and clustering analysis for revealing the main topics. Similarly, centrality is assessed, where high centrality is deemed crucial in connecting nodes. Parameters were set as below: (1) Time slicing (2010–2023), year per slice, and each figure's pruning were changed based on the needs of the map; (2) term sources were selected; and (3) node type was selected at a time. Meanwhile, the R package bibliometrix was used to output the collaboration map of countries.

## Results

### Annual publication

**Figure 1:**
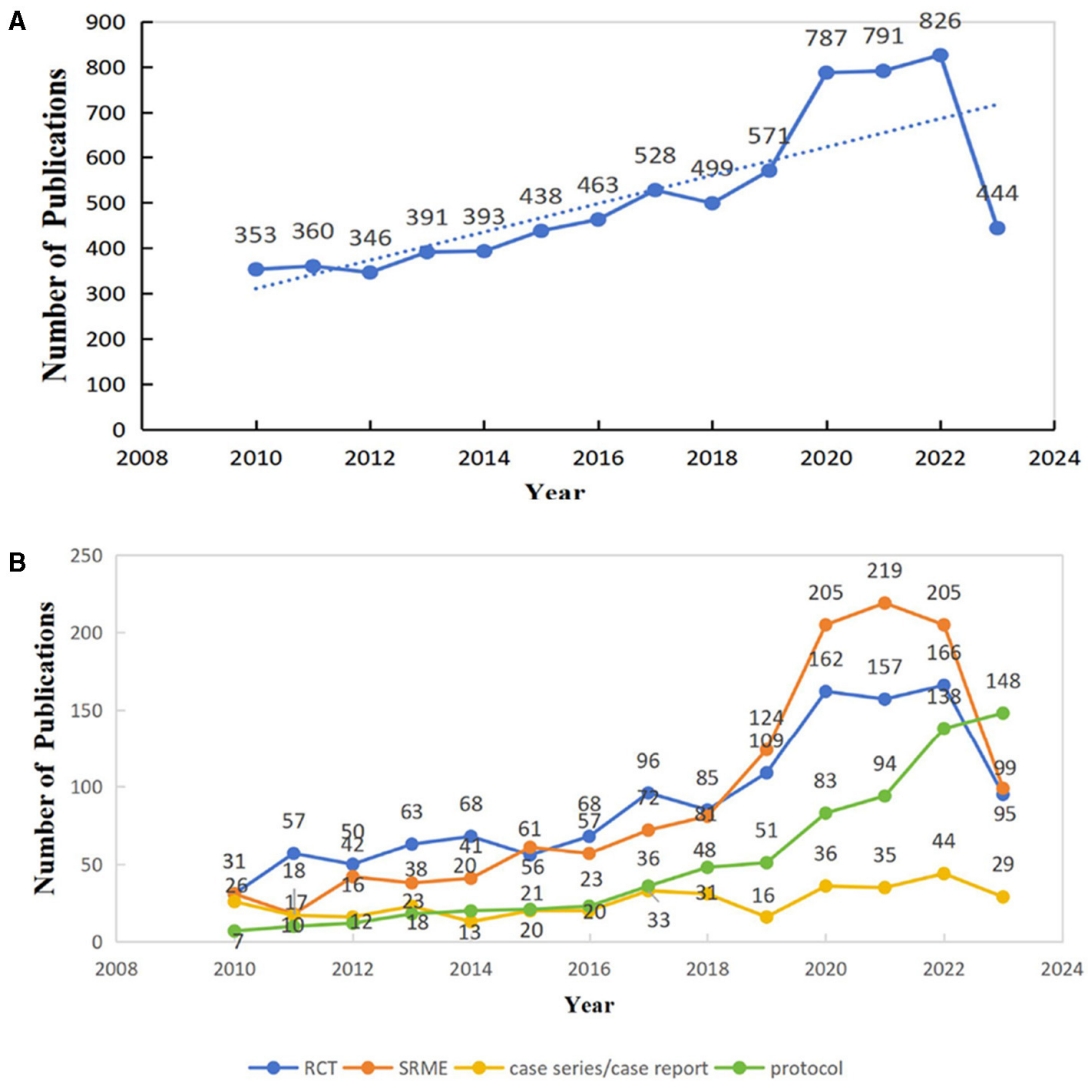
**(A)** Number of annual publications and growth trends of acupuncture analgesia in clinical practice globally. **(B)** Main study design trends in published clinical studies of acupuncture analgesia.SRME, systematic review and meta; RCT, randomized clinical trial.

From 1 January, 2010 to 1 September, 2023, a total of 7,190 documents were analyzed in our study. Of these, 5,226 (73%) were articles and 1,964 (27%) were reviews, resulting in an annual average of 553 publications. The publications on acupuncture analgesia in clinical settings exhibited a consistent growth trend (Figure 1A). Notably, between 2010 and 2012, the number of annual publications remained relatively stable. However, starting from 2012 onward, there was a gradual and sustained increase, surpassing 500 papers by 2017. While there was a slight decrease in 2018, the overall trend depicted robust growth, peaking at 826 in 2022 (Figure 1B).

Since 2010, there has been a consistent year-on-year increase in the number of publications across all research types. This trend is particularly notable in the of systematic review and meta (SRME) of clinical studies, as well as the numbers of RCTs, case reports/case series, and study protocols. Although the data from 2023 do not cover a full year, the trend indicates the continued use of acupuncture as a complementary and alternative therapy for analgesia in clinical settings.

### Distribution of countries/regions and institutions

Over the past 13 years, a total of 6,581 research institutions from 108 countries or regions published articles. Collaboration patterns among major countries or regions in acupuncture analgesia are shown in Supplementary Figures 2, 3. The result shows that China largely cooperated with the USA, England, South Korea, Italy, Australia, Singapore, and Japan. Supplementary Table 2 shows that most of the articles published were mainly derived from China (2,139 papers, 29.75%), followed by the USA (1,670 papers, 23.23%), South Korea (540 papers, 7.51%), England (476 papers, 6.62%), Australia (362 papers, 5.03%), and Canada (337 papers, 4.69%). In addition to the number of publications, centrality is considered a benchmark for judging research quality. Notably, the USA had the highest centrality of 0.21, emphasizing its pivotal role and significant influence in this field, followed by France (0.20) and Spain (0.15). China, with a slightly lower centrality (0.05), maintained an exceptional position in the collaboration network, fostering close ties with numerous countries.

The top 10 research institutions contributing 7,190 publications are listed in Supplementary Table 3. Half of these institutions were from China (5/10), followed by the USA (3/10) and South Korea (2/10). Chengdu University of Traditional Chinese Medicine (Chengdu Univ Chinese Med) published the most research papers (3.59%), followed by Beijing University of Chinese Medicine (Beijing Univ Chinese Med) (3.39%), Kyung Hee Univ, Harvard University (Harvard Univ), and Guangzhou University of Traditional Chinese Medicine (Guangzhou Univ Chinese Med). However, Harvard Univ had the highest centrality of 0.23, followed by Beijing Univ of Chinese Med (0.14), Harvard Medical School (0.13), University of Toronto (Univ Toronto) (0.10), and Univ California System (0.10). There was a close cooperative relationship among these institutions.

The institutions that published more than seven papers were analyzed using VOSviewer (Supplementary Figure 4), with 405 institutions included in the analysis network. Supplementary Figure 4B displays the distribution of institutions according to the average time of occurrence. Harvard Univ, Univ Toronto, Univ York, and Univ Oxford conducted relevant studies earlier. Meanwhile, research institutions represented by Chengdu Univ Chinese Med, Nanjing Univ of Chinese Med, Guangzhou Univ of Chinese Med, Tianjin Univ of Chinese Med, London South Bank Univ, Western Univ, Univ Hosp Zurich, and Univ Rey Juan Carlos started the research on acupuncture analgesia in clinical practice more recently. In addition, small-scale collaborations were established between some international institutions, but the low network nodes indicated a lack of global interagency collaboration. However, this finding does not fully explain the limited collaboration in the clinical application, highlighting the collaboration cannot be overstated.

### Analysis of authors and cited authors

The co-occurrence analysis conducted by the authors revealed the cooperation relationships among the authors, forming a co-occurrence map comprising 275 nodes and 461 links (Supplementary Figure 5A). These publications involved 32,676 authors, and Supplementary Table 4 lists the top 10 frequent authors in terms of publication volume and centrality. Liang FR from Chengdu Univ Chinese Med authored or co-authored the most articles (89 articles), followed by Li Y from the same institution (78 articles) and Wang Y from the China Institute of Guangzhou Univ Chinese Med (72 articles). However, centrality ranking was the most important. Lee MS, with a centrality of 0.13, ranked highest. As the founder of the International Society for Complementary Medicine Research and consultant to the Cochrane Collaboration in complementary and alternative medicine, Lee has published 66 articles in this field. Liang FR, the second-ranked author with a centrality of 0.12, serves as the vice president of the World Federation of Acupuncture and Moxibustion Societies, contributing extensive research on the clinical efficacy of acupuncture points for many years, and his articles covered many diseases, including chronic stable angina and migraine. Both authors have collaborated with others, but the level of collaboration between them is relatively low. Supplementary Figure 5A indicates a weak collaborative relationship among previous groups, forming numerous small-group collaborative networks. In recent years, an author from Spain has formed larger research groups but with less collaboration with mainstream groups internationally.

The co-citation map of the cited authors comprises 280 nodes and 2,342 links (Supplementary Figure 5B). MACPHERSON H was the most cited author (604 times), followed by Vickers AJ (510 times), Linde K (463 times), Moher D (380 times), and Witt CM (353 times). In terms of centrality, the top six cited authors were Vickers AJ (0.11), Han JS (0.11), Furlan AD (0.11), Macpherson H (0.09), Linde K (0.09), and Manheimer E (0.09). Clearly, they have demonstrated significant academic influence in the field. Notably, even though the articles written by the top-ranked cited authors were mostly published before 2020, they have been frequently cited in recent years, suggesting that acupuncture researchers are paying more attention to pain in clinical practice. Detailed information about the cited authors is shown in Supplementary Table 5.

### Analysis of journals and cited journals

In this study, a total of 1,483 journals have published papers in this field, with 69 journals that published more than 15 papers being selected for visualization (Supplementary Figure 6). The top 10 academic journals that published about 24.75% of the publications are enumerated in Supplementary Table 6. Sorted by publications, the most productive journal was *Medicine* (455 publications), followed by *Evid-Based Comple Alt Med* (289 publications) and then *Trials* (192 publications). According to the Journal Citation Reports of 2022, the average impact factor (IF) of these top 10 journals was 3.65. Among them, the leading journal with the highest IF (8.40) and highest H-index (244) was *Cochrane Db Syst Rev*, indicating its significant influence in the field of acupuncture analgesia. Of note, these journals were mostly located in the USA or England.

Supplementary Figure 6B presents a co-citation analysis of 590 journals, with a threshold of 80 citations from 29,341 journals. Seven clusters corresponding to seven colors in the figure highlight specific areas with varying clinical focus. Supplementary Table 7 outlines the top five journals in terms of frequency, which were more than 1,400, and *PAIN* had the most frequency (2,473) and citations (6,714), followed by *Cochrane Db Syst Rev, BMJ-Brit Med J, Evid-Based Comple Alt Med*, and *Acup Med*, and the top five journals in terms of centrality were *PAIN, Cochrane Db Syst Rev, BMJ-Brit Med J*, and *J Altern Comple Med*. In an analysis of co-citation and centrality, *PAIN* was identified as the core journal for acupuncture analgesia in clinical practice, and its published articles reflect the fundamentals of the research field.

### Keyword analysis

**Figure 2:**
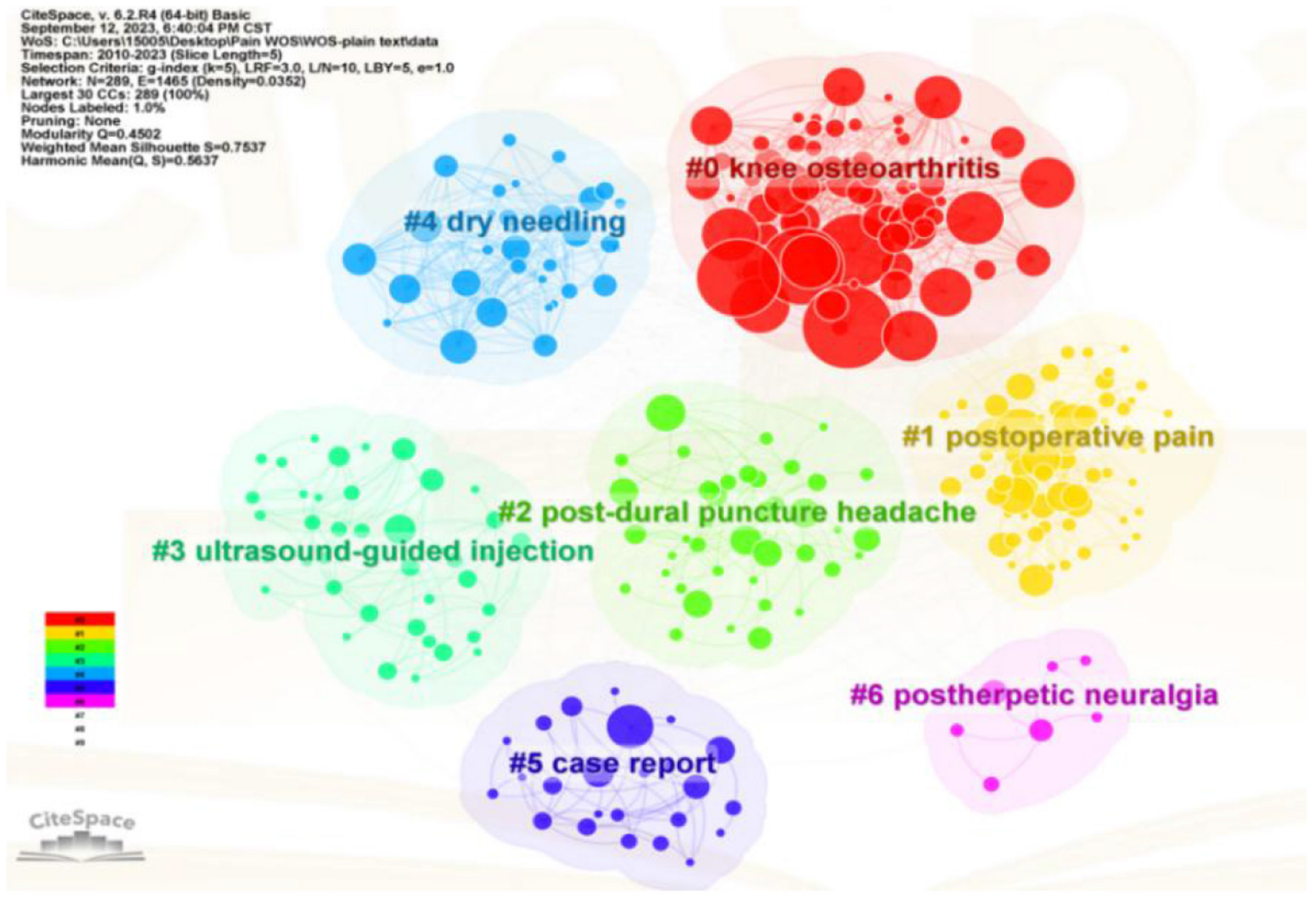
Visualization of keyword cluster analysis co-occurrence. In the co-occurrence network map, the nodes indicate the corresponding keywords, the size of the nodes denotes the number of articles containing the keywords, and the connecting lines between the nodes indicate the relationship between the keywords.

Keyword co-occurrence analysis can be performed to echo research themes, reflect research hotspots, and monitor frontier shifts in a field. Furthermore, cluster analysis provides a more holistic picture of the structure and evolution. In this study, all keywords were classified into seven clusters by CiteSpace (Figure 2): Cluster 0: “Knee osteoarthritis (red),” Cluster 1: “Postoperative pain (yellow),” Cluster 2: “Post-dural puncture headache (fluorescent green),” Cluster 3: “Ultrasound-guided injection (green),” Cluster 4: “Dry needling (blue),” Cluster 5: “Case report (indigo blue),” and Cluster 6: “Post-herpetic neuralgia (purple).” The magnitude of the keyword circle within each cluster was commensurate with the frequency of its appearance. The connecting links between each keyword were intricate, suggesting a complex connection between them. The mean silhouette was used to evaluate the clusters. The total silhouette value exceeded 0.7 (>0.5), implying a high degree of credibility in the obtained outcomes. The modularity (Q) was 0.4502 (>0.3), indicating that the clustering structure was substantial. The S values of the total silhouette were 0.7537, suggesting that the distribution and homogeneity of the clusters were well-defined, and the cluster was believed to be highly effective and persuasive.

**Figure 3:**
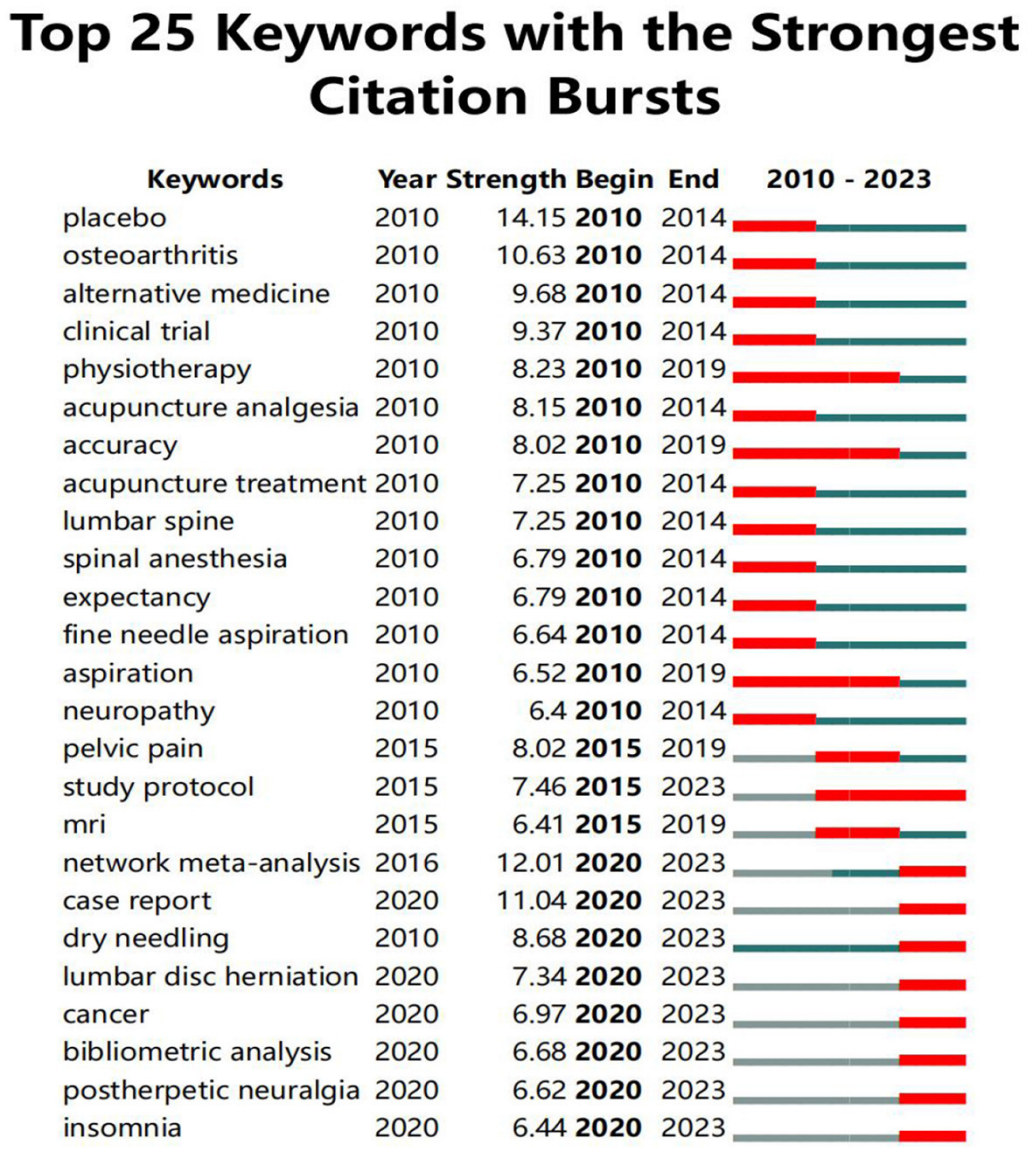
Top 25 keywords with the strongest citation bursts of acupuncture analgesia in clinical practice. The green line means the whole period, and the period during which a keyword's burst was identified is shown by the red line.

High-frequency keywords showed a popular theme, while high-centrality keywords reflected the status and importance of the corresponding research content in this field. As listed in Table 1, the top ten high-frequency keywords were as follows: “randomized controlled trial” (1,44), “management” (1,248), “pain” (1,052), “acupuncture” (1,020), “clinical trial” (672), “low back pain” (570), “therapy” (524), “systematic reviews” (512), “efficacy” (503), and “prevalence” (464). The top 10 high centrality keywords were as follows: “management” (0.29), “acupuncture” (0.16), “electro-acupuncture” (0.15), “pain” (0.12), “prevalence” (0.10), “low back pain” (0.08), “randomized clinical trial” (0.08), “diagnosis” (0.07), “tumors” (0.06), and “headache” (0.06). “Burst keywords” refer to keywords cited frequently over some time, indicating the frontier areas. The top 25 keywords of the citation burst are shown in Figure 3, and burst detection reveals important milestones in the field. The keywords “physiotherapy,” “accuracy,” and “aspiration” had a longer emergence time. In addition, the most significant and strongest citation burst belonged to “placebo.” Notably, since 2020, the keywords “network meta-analysis,” “case report,” “dry needling,” “lumbar disc herniation,” “cancer,” “bibliometric analysis,” “post-herpetic neuralgia,” and “insomnia” have been more prominently concentrated, indicating promising developments.

**Table 1:** Top 10 keywords related to the research of acupuncture analgesia in clinical practice.

**Ranking**	**Occurrences**	**Keywords**	**Ranking**	**Centrality**	**Keywords**
1	1,447	Randomized controlled trial	1	0.30	Management
2	1,248	Management	2	0.16	Acupuncture
3	1,052	Pain	3	0.15	Electro-acupuncture
4	1,020	Acupuncture	4	0.12	Pain
5	672	Clinical trial	5	0.10	Prevalence
6	570	Low back pain	6	0.08	Low back pain
7	524	Therapy	7	0.08	Randomized controlled trial
8	512	Systematic reviews	8	0.07	Diagnosis
9	503	Efficacy	9	0.06	Tumors
10	464	Prevalence	10	0.06	Headache

**Figure 4:**
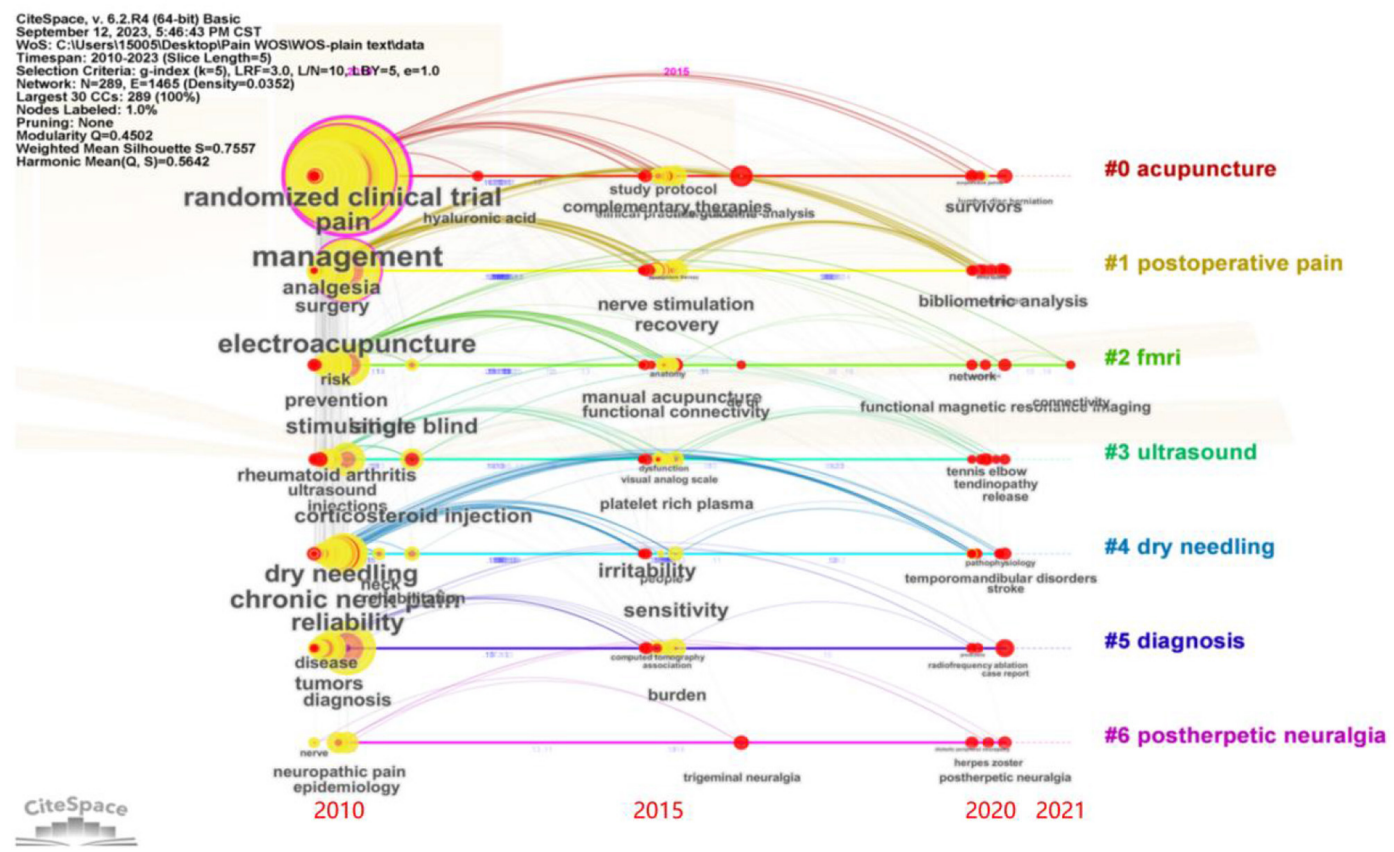
Timeline diagram of keywords in the field of acupuncture analgesia in clinical practice. The color-coded curves denote co-citation connections formed within specific years, the large nodes, or those with red halos—for their notable citation metrics or sudden increases in citations. Below these timelines, the year's top three cited works are shown, with the highest-cited work at the bottom.

Based on the cluster map, timeline diagrams could illustrate the panorama, the historical evolution, and the frontiers of hotspots in the research field over the last 13 years. The keyword clustering of research continued up to the present and mainly involved #0 acupuncture, #1 postoperative pain, #2 FMRI, #3 ultrasound, #4 dry needling, #5 diagnose, and #6 post-herpetic neuralgia. As displayed in Figure 4, timeline visualizations organize clusters horizontally, mapping each from left to right according to publication dates, displayed at the visualization's bottom edge in CiteSpace. The arrangement of clusters follows a vertical, size-based descending order. The keywords in this field from 2010 to 2023 focused on randomized clinical trials, pain, management, electro-acupuncture, dry needling, tumors, and neuropathic pain, which were the most basic and important research directions. In recent years, keywords such as survivors, network, tennis elbow, and post-herpetic neuralgia have appeared. The multidisciplinary intersection such as bibliometrics is the current hotspot and trend of scientific research, which will significantly promote the development of the depth and breadth of scientific research.

### Cited references analysis

**Figure 5:**
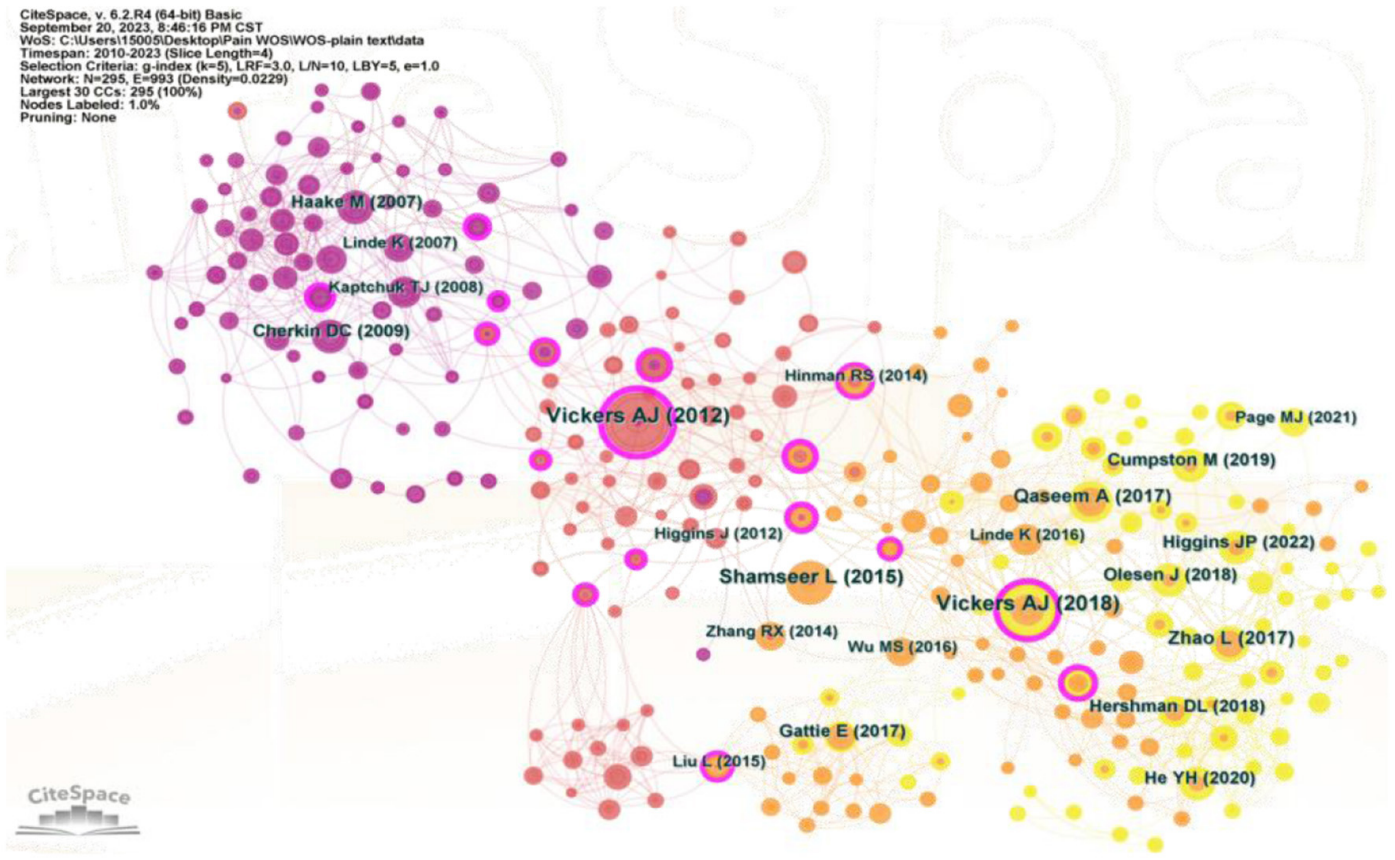
Visualization of co-cited references. Each node represents a paper, the node size indicates the number of times that the paper has been cited, and the links between nodes reflect the strength of co-citations, while the node color indicates the citation year.

Co-citation analysis is crucial for identifying the key literature. A total of 186,873 cited references from the 7,190 publications were analyzed as co-cited references. Figure 5 displays the clinical acupuncture analgesia network with 295 nodes and 993 links. Table 2 shows the top 10 cited references sorted by the number of citations. These references were landmark references in the field and set the stage for future research. These references, spanning from 2009 to 2020, include three clinical trials, three systematic reviews and meta-analyses, two guidelines or guidance, one PRISMA statement, and one on headache disorders classification.

**Table 2:** The top 10 highly cited papers in the field of acupuncture analgesia in clinical practice (2010–2023).

**Rank**	**Counts**	**Title**	**Author**	**Journal**	**Publication year**	**Centrality**	**2022 IF**	**DOI**
1	156	Acupuncture for chronic pain: individual patient data meta-analysis	Vickers et al. (18)	Arch Intern Med	2012	0.46	38.99	10.1001/archinternmed.2012.3654
2	129	Acupuncture for chronic pain: update of an individual patient data meta-analysis	Vickers et al. (19)	J Pain	2018	0.34	4.00	10.1016/j.jpain.2017.11.005
3	93	Preferred reporting items for systematic review and meta-analysis protocols (PRISMA-P) 2015 statement	Shamseer et al. (20)	Systematic Reviews	2015	0.00	3.70	10.1186/2046-4053-4-1
4	79	Noninvasive treatments for acute, subacute, and chronic low back pain: a clinical practice guideline from the American college of physicians	Qaseem et al.	Ann Intern Med	2017	0.09	39.20	10.7326/M16-2367
5	73	The long-term effect of acupuncture for migraine prophylaxis: a randomized clinical trial	Zhao et al. (21)	Jama Intern Med	2017	0.06	38.99	10.1001/jamainternmed.2016.9378
6	62	Headache classification committee of the international headache society (IHS) the international classification of headache disorders, 3rd edition	Olesen J	Cephalalgia	2018	0.03	4.90	10.1177/0333102417738202
7	62	A randomized trial comparing acupuncture, simulated acupuncture, and usual care for chronic low back pain	Cherkin et al. (22)	Arch Intern Med	2009	0.04	38.99	10.1001/archinternmed.2009.65
8	61	Updated guidance for trusted systematic reviews: a new edition of the Cochrane Handbook for Systematic Reviews of Interventions	Cumpston et al. (23)	Cochrane Db Syst Rev	2019	0.03	8.40	10.1002/14651858.ED000142
9	58	Clinical evidence for association of acupuncture and acupressure with improved cancer pain: a systematic review and meta-analysis	He et al. (24)	Jama Oncol	2020	0.07	28.40	10.1001/jamaoncol.2019.5233
10	57	Effect of acupuncture vs. sham acupuncture or waitlist control on joint pain related to aromatase inhibitors among women with early-stage breast cancer: a randomized clinical trial	Hershman et al. (25)	Jama-J Am Med Assoc	2018	0.09	120.70	10.1001/jama.2018.8907

Arch Intern Med has been renamed Jama Intern Med.

Vickers et al. (18) article stood out with 156 co-citations and the highest centrality (0.46). Focusing on acupuncture for chronic pain, it emphasized the efficacy of acupuncture beyond a placebo, providing clarity on its clinical utility. In addition, this article is an excellent demonstration in clarifying the utility of acupuncture analgesia in clinical practice. Notably, Vickers AJ' s meta-analysis update ranked second in citations and centrality, suggesting that the authors and their two articles had a dominant influence in the field. Detailed data of the remaining highly cited articles are listed in Table 2.

## Discussion

This study investigated the literature on clinical acupuncture analgesia, employing the bibliometric analysis to characterize publications, analyze co-occurrences and clustering, and reveal current landscapes and frontier topics. As the first bibliometric study in this area, it aims to guide future research directions.

### Basic information

From 2010, the output of annual publications related to acupuncture analgesia in clinical practice has steadily increased. The number of publications in 2022 was the highest, accounting for 11.49% of all publications, which suggests a growing research interest in this field. Two main reasons contribute to this phenomenon: the evolving focus of modern medicine on patients' quality of life (26) and the proven clinical efficacy of acupuncture analgesia in reducing drug dosages and adverse effects and serving as an opioid alternative (24, 27). Research conducted between 2010 and 2023 in China has shown rapid growth, surpassing the USA in a number of publications after 2018, while in other countries or regions, the number of annual publications was basically stable, indicating that research has gradually shifted from the USA, the acknowledged center of the Western world, to China, the central power of the Eastern world. Despite China leading in the production of published papers, the USA maintains higher centrality (0.21 vs. 0.05), highlighting its continued dominance in acupuncture analgesia research. The UK, Germany, and Canada, as major research powers, exhibit well-established collaboration networks, emphasizing their substantial contributions to international cooperation. This finding suggested the growing acceptance of acupuncture as a complementary therapy. Conversely, despite China and South Korea having a long history with acupuncture and numerous clinical trials (28), their actual contribution and influence are relatively limited.

In the institutional cooperation network, Chengdu Univ Chinese Med, Beijing Univ Chinese Med, and Kyung Hee Univ stood out for their comprehensive strength. Chengdu Univ Chinese Med and Beijing Univ Chinese Med, as pioneers in Chinese medicine, were vital medical innovation research bases and have trained a large number of professional medical and health talents in acupuncture and moxibustion. Kyung Hee Univ, a research-centered university, has leading research institutions and academic centers worldwide. As early as 1976, The Korean Medicine Hospital of Kyung Hee University was the first research institution in the world to successfully conduct acupuncture anesthesia for cesarean operations and held the first World Congress of Acupuncture, which had high authority.

However, Harvard Univ held the most centrality globally, reflecting its influence in acupuncture analgesia and the broader medical field. China and its relevant scientific institutions still need to improve publication quality and international cooperation to boost influence. Most noteworthy is the fact that there was no institution from the UK and Australia, among the top five countries with the most publications, listed in the top 10 list, possibly because the institutions conducting research for acupuncture anesthesia in those two countries were relatively fragmented and the support base for cooperation was also not enough, so domestic collaboration between agencies was more important than international collaboration. International collaboration is currently insufficient, necessitating improved communication and cooperation.

Co-authorship analysis indicated communication between academics in the research area, which is an important indicator of scholarly collaboration (12–16). Our analysis of authors and organizational affiliations revealed a significant collaboration among Asian researchers in acupuncture analgesia. The top 10 authors, all contributing over 50 articles, included Liang FR (89 articles) from Chengdu Univ Chinese Med, a key figure in this field. In terms of centrality, among the top five most cited authors, three authors, namely, Liang FR, Lao LX, and Zheng H, were high-impact authors from China. In terms of the authors' cooperation network, there were multiple mature cooperation teams with a certain degree of cooperation. However, the cooperation was relatively less stable, which indicated that cooperation between prolific authors was limited to smaller groups, and they tended to work with stable collaborative teams and were largely confined to their respective institutions.

Through the analysis of co-cited authors, the top five cited authors with the most citations (2,310 times) were from Europe and the USA, such as Macpherson H (England), Vickers AJ (USA), LindeI K (Germany), Moher D (Canada), and Witt CM (Germany), while those published by Vickers AJ (USA), Han JS (China), Furlan AD (Canada), Macpherson H (England), and Linde K (Germany) had the highest centrality in articles. MacPherson H, as the first author, was a “core strength” researcher in this field. In addition, he focused on the evaluation of the effectiveness and safety of acupuncture and the neuroimaging research on the mechanism of acupuncture. For example, MacPherson et al. presented an updated reporting guideline in 2010, which stood for *Revised Standards for Reporting Interventions in Clinical Trials of Acupuncture* (29). Citations might not be the best index of publication quality; although a considerable number of articles were from Asia, improvement in the quality of these articles was necessary.

Through the analysis of these journals, we found that the top 10 academic journals published 1,773 articles, accounting for 25% of all articles, and the results of this analysis suggested that these journals had a strong interest in articles regarding acupuncture analgesia in clinical practice but is different from the findings of the previous study, which may be due to the limitation of the study pertaining to the field of acupuncture analgesia (30). *MEDICINE* was the most critical journal and has made significant contributions in this field. Although *COCHRANE DB SYST REV* ranked fifth with only 130 publications, it ranked second for citations with the highest IF (8.40) and H-index (273), indicating that this journal was very influential in this field and was worth learning for scholars. Notably, the 10 journals with the highest outputs, with the exception of *COCHRANE DB SYST REV*, generally had low impact factors (average IF < 3) but still had a number of articles that received a high number of citations. Most of these journals were relevant to complementary alternative therapies and pain research. However, publishing clinical studies on acupuncture analgesia in high-quality journals is still a challenge. According to the analysis of the cited journals, the top-ranking journal for both frequency and centrality was *PAIN*. These data will help future scholars to select appropriate journals to reference or submit articles in related fields.

### Research frontiers and trends

Keywords and references can reflect the content of the research, which is helpful in identifying hotspots and frontiers from their frequency, centrality, and clustering distribution (31). From cluster-related topics, we can identify the current frontiers from the perspective of research areas; knee osteoarthritis (32), postoperative pain (33), post-dural puncture headache (34, 35), dry needling (36), case report (35, 37), and post-herpetic neuralgia (38) are the areas of the main research focus, which shows that researchers are highly interested in acupuncture analgesia for different disease types. In addition, a burst keyword can indicate cutting-edge research topics and reveal studies that have potential or are of interest. Since 2020, the burst keywords used were “network meta-analysis,” “case report,” “dry needling,” “lumbar disc herniation,” “cancer,” “bibliometric analysis,” “post-herpetic neuralgia,” and “insomnia,” indicating that researchers currently focus on those promising developments. The researchers are interested in case reports on acupuncture analgesia, exploring the analgesic effects of different types of acupuncture (dry needling); relieving pain-induced adverse effects, such as insomnia; and incorporating a cross-disciplinary (bibliometric analysis) approach to exploring future directions in the treatment of pain with acupuncture. From 2015, this protocol has gradually become a hotspot. We believe the design and improvement of protocols on acupuncture will be hot topics of research in the future (20, 39–42).

High-frequency keywords and burst keywords (RCTs and clinical trials) demonstrated that researchers are very interested in verification of the effectiveness of acupuncture analgesia in clinical practice, and the effectiveness has been the focus of this research area. Through visualization of co-cited references, and as observed in the highly cited paper, some guidelines recommending acupuncture for chronic pain are the most cited, which also suggests that the effectiveness of acupuncture for chronic pain remains the focus of researchers in this field (43, 44). The top 1 reference by frequency and centrality is a meta-analysis of individual patient data on acupuncture for chronic pain, which confirms the effectiveness of acupuncture for lower back and neck pain, osteoarthritis, chronic headache, and shoulder pain (18). Of note, the top three highly cited papers were all related to meta-analysis. This result suggests that researchers have gone beyond looking for evidence of the effectiveness of acupuncture from RCTs or trials, both by looking for high-quality evidence via evidence-based medicine and by following standard guidelines for reporting interventions in acupuncture clinical trials to implement meta-analyses on acupuncture analgesia (20).

### Strengths and limitations

In this study, we employed a more comprehensive search strategy to systematically organize the subject terms involving acupuncture, which was not available in previous bibliometrics related to acupuncture, to avoid the impact of omitted literature on the results of the study. Second, by using bibliometrics, we performed a visual analysis of literature, provided a channel for researchers to summarize research status and key research forces, and predict the development trends in the field. In addition, this study distinguished itself from previous studies limited to a single field of acupuncture analgesia, adding value for clinical researchers. To our knowledge, this is the first bibliometric analysis of acupuncture analgesia in clinical practice. Moreover, various methods are used to analyze data, allowing for a multidimensional interpretation of conclusions and offering insights into potential global research collaborations.

First, our search was restricted to English-language publications, as non-English articles constitute a small percentage (about 2%) of total articles in WoS. It is expected that the overall trends of our results might be similar to the results without language restrictions. As one of the most authoritative scientific and technological literature retrieval tools, WoS could not cover all the research on acupuncture analgesia in clinical practice. The journals included in the SCI-E of WoS database are described as world-leading journals due to a rigorous selection process. Thus, publications in WoS still can be a representative of research in the discipline. Second, despite efforts to incorporate numerous search terms, the study might have overlooked some terms, potentially neglecting the latest research trends. Third, the study did not include papers published after the search date due to the continuous updates to the database, resulting in potential gaps in literature retrieval. In addition, the number of clusters and the label of clusters in the network analysis will vary depending on the resolution of clustering and the subjective views of the authors. Finally, affiliations may not precisely differentiate associated organizations; for instance, Harvard Med School and Harvard Univ are separately analyzed.

## Conclusion

In conclusion, acupuncture analgesia is valuable for research and clinical applications. Offering a comprehensive overview from 2010 to 2023, the findings serve as a valuable reference for potential collaborations and highlight opportunities for future developments in this field. The findings indicate a rapid expansion, with China leading in publication numbers and the USA demonstrates greater influence in this field. However, limited collaboration between countries and institutions may hinder progress. Increased cooperation and data exchange among institutions and scholars are essential, contributing to the further expansion and international acceptance of acupuncture.

## Data availability statement

The original contributions presented in the study are included in the article/Supplementary material, further inquiries can be directed to the corresponding author.

## Author contributions

Z-QL: Conceptualization, Data curation, Formal analysis, Investigation, Methodology, Writing—original draft, Writing—review & editing. X-FW: Conceptualization, Data curation, Formal analysis, Investigation, Writing—original draft. CF: Conceptualization, Data curation, Formal analysis, Writing—original draft. Y-TF: Conceptualization, Methodology, Supervision, Writing—review & editing. J-PL: Conceptualization, Formal analysis, Funding acquisition, Writing—review & editing.
