# Supplementary material for: Global trends of acupuncture clinical research on analgesia from 2010 to 2023: a bibliometric and visualization analysis
Source: Front Neurol. 2024 Apr 11;15:1368988. doi: 10.3389/fneur.2024.1368988 (PMC11043534; doi:10.3389/fneur.2024.1368988)
Supplement: Supplementary file 1 [file Data_Sheet_1.docx]

**Global Trends of Acupuncture Clinical Research on Analgesia from 2010 to 2023: A Bibliometric and Visualization Analysis**

Supplementary Table 1.The Topic Search Query(SCI-expanded of WoS)

Supplementary Table 2: Top 10 countries/regions related to the research of acupuncture analgesia in clinical

Supplementary Table 3: Top 10 institutions related to the research of acupuncture analgesia in clinical

Supplementary Table 4: Top 10 Authors related to the research of acupuncture analgesia in clinical

Supplementary Table 5: Top 10 cited authors related to the research of acupuncture analgesia in clinical

Supplementary Table 6: Top 10 Journals with the Highest Frequency Values for Studies on acupuncture analgesia in clinical

Supplementary Table 7: Top 10 Cited Journals with the Highest Frequency Values for acupuncture analgesia in clinical

Supplementary Figure 1: Study Flow Chart.TS indicates topic search.

Supplementary Figure 2: Cooperative relationships between major countries or regions of acupuncture analgesia in clinical.

Supplementary Figure 3: Cooperative relationships between different countries or regions of acupuncture analgesia in clinical.

Supplementary Figure 4: Map of institutions’ collaborations of acupuncture analgesia in clinical.

Supplementary Figure 5:Visualization of the author and co-cited author in the field of acupuncture analgesia in clinical.

Supplementary Figure 6: (A) Map of journals producing articles.(B)Map of co-cited journals with articles.

| **Supplementary Table 1.The Topic Search Query(SCI-expanded of WoS) 2010-01-01-----2023.09-01** | | |
| --- | --- | --- |
| **Set** | **Results** | **Search Query** |
| #1 | 108,354 | ((((((((((((((((((((((((((((((((((((((((((((((((((((((((((((((((((((((TS=(Acupuncture Therapy)) OR TS=(Acupuncture Treatment)) OR TS=(Pharmacoacupuncture Treatment)) OR TS=(Acupuncture Analgesia)) OR TS=(Acupotomy)) OR TS=(Acupuncture)) OR TS=(acupunct*)) OR TS=(Pharmaco-acupuncture)) OR TS=(pharmacupuncture)) OR TS=(Electro-acupuncture)) OR TS=(electroacupunct*)) OR TS=(electro-acupunct*) OR TS=(Transcutaneous electrical acupoint stimulation)) OR TS=(transcutaneous electric nerve stimulation)) OR TS=(Acupuncture Point)) OR TS=(Body Acupuncture)) OR TS=(Manual Acupuncture)) OR TS=(Ear Acupuncture)) OR TS=(Acupuncture, Ear*)) OR TS=(auriculotherap*)) OR TS=(auriculoacupunct*)) OR TS=(Auricular Acupuncture)) OR TS=(Auricular acupoint penetration needling)) OR TS=(scalp acupuncture)) OR TS=(Auricular Needle)) OR TS=(Auricular point sticking)) OR TS=(Acupuncture Point*)) OR TS=(Acupoint)) OR TS=(Acupoint*)) OR TS=(Warm Acupuncture)) OR TS=(Warming needle moxibustion)) OR TS=(Wen acupuncture)) OR TS=(Warm-needle moxibustion)) OR TS=(Wet Acupuncture)) OR TS=(Sinew acupuncture)) OR TS=(Moxibustion)) OR TS=(Moxabustion Acupuncture)) OR TS=(Acupoint Injection)) OR TS=(Catgut embedding)) OR TS=(Skin acupuncture)) OR TS=(Wrist-ankle acupuncture)) OR TS=(Catgut implantation at acupoint)) OR TS=(Needling)) OR TS=(Needle Acupuncture)) OR TS=(Dry Needling)) OR TS=(Laser on Acupuncture Points)) OR TS=(laser acupuncture)) OR TS=(Fire needle)) OR TS=(Fire acupuncture)) OR TS=(Fire needlinge)) OR TS=(Heated Needle)) OR TS=(Filiform Needle)) OR TS=(plum blossom needle)) OR TS=(Intradermal needling)) OR TS=(Imbedding Needle)) OR TS=(Fire-needle)) OR TS=(Temperature needle)) OR TS=(Needle-Knife)) OR TS=(Internal Thermal Needle)) OR TS=(Acupoint Catgut Embedding)) OR TS=(Acupuncture Point Injection Therapy)) OR TS=(Ear Motional Acupuncture)) OR TS=(Eye acupuncture)) OR TS=(Fu’s acupuncture)) OR TS=(floating needling)) OR TS=( fu’s subcutaneous needling)) OR TS=(Ashi point)) OR TS=(Acupress*)) OR TS=(Shiatsu)) OR TS=(meridian-collateral theory)) OR TS=(meridian)); Timespan = 2010.01.01–2023.09.01 |
| #2 | 969,186 | (((((((((((((((((((((((((((((((((((((((((((((((((((TS=(Pain)) OR TS=(Pain*)) OR TS=(Acute Pain)) OR TS=(Chronic Pain)) OR TS=(ache)) OR TS=(*ache)) OR TS=(Headache)) OR TS=(Toothache)) OR TS=(Stomachache)) OR TS=(Backache)) OR TS=(Waistache)) OR TS=(Earache)) OR TS=(Sore)) OR TS=(Sore*)) OR TS=(cramp*)) OR TS=(sprain)) OR TS=(injuri*)) OR TS=(Causalgia))) OR TS=(Neuralgia)) OR TS=(Somatosensory Disorders)) OR TS=(Reflex Sympathetic Dystrophy)) OR TS=(Hereditary Sensory and Autonomic Neuropathies)) OR TS=(Cystitis, Interstitial)) OR TS=(Nociceptors)) OR TS=(Myalgia)) OR TS=(Mastodynia)) OR TS=(Vulvodynia)) OR TS=(Arthralgia)) OR TS=(Fibromyalgia)) OR TS=(Dysmenorrhea)) OR TS=(Temporomandibular Joint Dysfunction Syndrome)) OR TS=(Somatoform Disorders)) OR TS=(Prostatitis)) OR TS=(Migraine)) OR TS=(Carpal tunnel syndrome)) OR TS=(Osteoarthritis)) OR TS=(Irritable bowel syndrome)) OR TS=(gout)) OR TS=(Arthrolithiasis)) OR TS=(Angina pectoris)) OR TS=(Sciatica)) OR TS=(Colic)) OR TS=(Colic*)) OR TS=(Cramp*)) OR TS=(prick*)) OR TS=(analges*)) OR TS=(nocicept*)) OR TS=(neuropath*)) OR TS=(Ankylosing spondylitis)) OR TS=(Herpes)); Timespan = 2010.01.01–2023.09.01 |
| #3 | 2,747,929 | (((((TS=(RCT)) OR TS=(Clinical Trial*)) OR TS=(Randomized controlled trial)) OR TS=(Clinical)) OR TS=(Clinic Trial*)); Timespan = 2010.01.01–2023.09.01 |
| #4 | 8,118 | #1 AND #2 AND #3 |

| **Supplementary Table 2. Top 10 countries/regions related to the research of acupuncture analgesia in clinical** | | | | | | |
| --- | --- | --- | --- | --- | --- | --- |
| **Ranking** | **Publications** | **Country/Region** | **Percentage(n/7190)** | **Ranking** | **Centrality** | **Country/Region** |
| **1** | 2139 | CHINA | 29.75 | **1** | 0.21 | USA |
| **2** | 1670 | USA | 23.23 | **2** | 0.20 | FRANCE |
| **3** | 540 | SOUTH KOREA | 7.51 | **3** | 0.15 | SPAIN |
| **4** | 476 | ENGLAND | 6.62 | **4** | 0.14 | ENGLAND |
| **5** | 362 | AUSTRALIA | 5.03 | **5** | 0.13 | ITALY |
| **6** | 337 | CANADA | 4.69 | **6** | 0.11 | AUSTRALIA |
| **7** | 299 | GERMANY | 4.16 | **7** | 0.09 | SWITZERLAND |
| **8** | 290 | SPAIN | 4.03 | **8** | 0.08 | CANADA |
| **9** | 276 | ITALY | 3.84 | **9** | 0.07 | GERMANY |
| **10** | 252 | BRAZIL | 3.50 | **10** | 0.05 | CHINA |

| **Supplementary Table 3. Top 10 institutions related to the research of acupuncture analgesia in clinical** | | | | | | |
| --- | --- | --- | --- | --- | --- | --- |
| **Ranking** | **Publications** | **Institutions** | **Percentage(n/7190)** | **Ranking** | **Centrality** | **Institutions** |
| **1** | 258 | Chengdu University of Traditional Chinese Medicine | 3.59 | **1** | 0.23 | Harvard University |
| **2** | 244 | Beijing University of Chinese Medicine | 3.39 | **2** | 0.14 | Beijing University of Chinese Medicine |
| **3** | 211 | Kyung Hee University | 2.94 | **3** | 0.13 | Harvard Medical School |
| **4** | 187 | Harvard University | 2.60 | **4** | 0.10 | University of Toronto |
| **5** | 151 | Guangzhou University of Chinese Medicine | 2.10 | **5** | 0.10 | University of California System |
| **6** | 150 | China Academy of Chinese Medical Sciences | 2.08 | **6** | 0.08 | Pennsylvania Commonwealth System of Higher Education |
| **7** | 130 | Korea Institute of Oriental Medicine | 1.81 | **7** | 0.08 | University of London |
| **8** | 129 | Capital Medical University | 1.794 | **8** | 0.08 | Universidad Rey Juan Carlos |
| **9** | 125 | Harvard Medical School | 1.739 | **9** | 0.07 | Chengdu University of Traditional Chinese Medicine |
| **10** | 117 | University of California System | 1.627 | **10** | 0.07 | University System of Maryland |

| **Supplementary Table 4.Top 10 Authors related to the research of acupuncture analgesia in clinical** | | | | | | | |
| --- | --- | --- | --- | --- | --- | --- | --- |
| **Ranking** | **Publications** | **Author** | **Country/Region** | **Ranking** | **Centrality** | **Author** | **Country/Region** |
| **1** | 89 | Liang FR | China | **1** | 0.13 | Lee MS | South Korea |
| **2** | 78 | Li Y | China | **2** | 0.12 | Liang FR | China |
| **3** | 72 | Wang Y | China | **3** | 0.11 | Lao LX | China |
| **4** | 70 | Li J | China | **4** | 0.11 | Kim Tae-Hun | South Korea |
| **5** | 66 | Lee MS | South Korea | **5** | 0.11 | Zheng H | China |
| **6** | 60 | Fernandez-de-las-penas C | Spain | **6** | 0.10 | Li Y | China |
| **7** | 60 | Zhao L | China | **7** | 0.09 | Zhao L | China |
| **8** | 54 | Chen J | China | **8** | 0.09 | Macpherson H | England |
| **9** | 54 | Lee JH | South Korea | **9** | 0.07 | Witt CM | Germany |
| **10** | 54 | Liu CZ | China | **10** | 0.05 | Ernst E | England |

| **Supplementary Table 5.Top 10 cited authors related to the research of acupuncture analgesia in clinical** | | | | | | | |
| --- | --- | --- | --- | --- | --- | --- | --- |
| **Ranking** | **Publications** | **Author** | **Country/Region** | **Ranking** | **Centrality** | **Author** | **Country/Region** |
| **1** | 604 | Macpherson H | England | **1** | 0.11 | Vickers AJ | USA |
| **2** | 510 | Vickers AJ | USA | **2** | 0.11 | Han JS | China |
| **3** | 463 | LindeI K | Germany | **3** | 0.11 | Furlan AD | Canada |
| **4** | 380 | Moher D | Canada | **4** | 0.09 | Macpherson H | England |
| **5** | 353 | Witt CM | Germany | **5** | 0.09 | Linde K | Germany |
| **6** | 345 | Kaptchuk TJ | USA | **6** | 0.09 | Manheimer E | USA |
| **7** | 324 | Han JS | China | **7** | 0.08 | Kaptchuk TJ | USA |
| **8** | 321 | Higgins JPT | England | **8** | 0.07 | Chou R | USA |
| **9** | 305 | Schulz KF | USA | **9** | 0.06 | Witt CM | Germany |
| **10** | 303 | Ernst E | England | **10** | 0.06 | Kong J | China |

| **Supplementary Table 6. Top 10 Journals with the Highest Frequency Values for Studies on acupuncture analgesia in clinical** | | | | | |
| --- | --- | --- | --- | --- | --- |
| **Ranking** | **Journal** | **Frequency** | **Country** | **IF (2022)** | **JCR** |
| **1** | MEDICINE | 455 | USA | 1.600 | Q3 |
| **2** | EVID-BASED COMPL ALT | 289 | USA | NA | NA |
| **3** | TRIALS | 192 | England | 2.5000 | Q2 |
| **4** | ACUPUNCTURE IN MEDICINE | 173 | England | 2.5000 | Q2 |
| **5** | COCHRANE DB SYST REV | 130 | England | 8.4001 | Q1 |
| **6** | J ALTERN COMPLEM MED | 111 | USA | 2.6002 | Q2 |
| **7** | PAIN MEDICINE | 109 | England | 3.1000 | Q2 |
| **8** | BMJ OPEN | 108 | England | 2.8997 | Q2 |
| **9** | JOURNAL OF PAIN RESEARCH | 107 | USA | 2.7001 | Q3 |
| **10** | PLOS ONE | 99 | USA | 3.7001 | Q2 |
| **Abbreviations:** EVIDENCE BASED COMPLEMENTARY AND ALTERNATIVE MEDICINE, EVID-BASED COMPL ALT ; JOURNAL OF ALTERNATIVE AND COMPLEMENTARY MEDICINE, J ALTERN COMPLEM MED; COCHRANE DATABASE OF SYSTEMATIC REVIEWS, COCHRANE DB SYST REV. The journal (EVID-BASED COMPL ALT)has been dropped from the SCI | | | | | |

| **Supplementary Table 7. Top 10 Cited Journals with the Highest Frequency Values for acupuncture analgesia in clinical** | | | | | | | |
| --- | --- | --- | --- | --- | --- | --- | --- |
| **Ranking** | **Journal** | **Frequency** | **Citation** | **Centrality** | **IF (2022)** | **Country** | **JCR** |
| **1** | PAIN | 2473 | 6714 | 0.20 | 7.4001 | USA | Q1 |
| **2** | COCHRANE DB SYST REV | 2073 | 4433 | 0.16 | 8.4001 | England | Q1 |
| **3** | BMJ-BRIT MED J | 1914 | 3146 | 0.11 | 105.7030 | England | Q1 |
| **4** | EVID-BASED COMPL ALT | 1875 | 3747 | 0.09 | NA | USA | NA |
| **5** | ACUPUNCTURE IN MEDICINE | 1757 | 3643 | 0.10 | 2.5000 | England | Q2 |
| **6** | LANCET | 1714 | 2551 | 0.03 | 168.9015 | England | Q1 |
| **7** | J ALTERN COMPLEM MED | 1703 | 3320 | 0.14 | 2.6002 | USA | Q2 |
| **8** | JAMA-J AM MED ASSOC | 1603 | 2287 | 0.04 | 120.7003 | USA | Q1 |
| **9** | ANN INTERN MED | 1469 | 2289 | 0.05 | 39.2001 | USA | Q1 |
| **10** | PLOS ONE | 1440 | 2203 | 0.02 | 3.7001 | USA | Q2 |

Supplementary Figure 1. Study Flow Chart.TS indicates topic search.

Supplementary Figure 2.Cooperative relationships between major countries **or regions** of acupuncture analgesia in clinical.

Supplementary Figure 3.Cooperative relationships between different countries or regions of acupuncture analgesia in clinical. (A) The network of different countries or regions visualization. (B) The distribution of countries or regions according to the average time of occurrence.

Supplementary Figure 4. Map of institutions’ collaborations of acupuncture analgesia in clinical. (A)The network of different institutions visualization. (B) The distribution of institutions according to the average time of occurrence.

Supplementary Figure 5. Visualization of the author and co-cited author in the field of acupuncture analgesia in clinical. (A) The collaborative relationships of authors. (B) The co-citation relationships of cited authors.

Supplementary Figure 6.(A) Map of journals producing articles.(B)Map of co-cited journals with articles.
